# The impact of diet, body composition, and physical activity on child bone mineral density at five years of age—findings from the ROLO Kids Study

**DOI:** 10.1007/s00431-019-03465-x

**Published:** 2019-11-01

**Authors:** Marco K. McVey, Aisling A. Geraghty, Eileen C. O’Brien, Malachi J. McKenna, Mark T. Kilbane, Rachel K. Crowley, Patrick J. Twomey, Fionnuala M. McAuliffe

**Affiliations:** 1 UCD Perinatal Research Centre, School of Medicine, University College Dublin, National Maternity Hospital, Dublin, Ireland; 2 Department of Endocrinology, St. Vincent’s University Hospital, Dublin, Ireland; 3 School of Medicine, University College Dublin, Dublin, Ireland; 4 Department of Clinical Chemistry, St. Vincent’s University Hospital, Dublin, Ireland

**Keywords:** Child bone health, Vitamin D, Calcium, Activity, Screen time, Child BMI

## Abstract

Bone health is extremely important in early childhood because children with low bone mineral density (BMD) are at a greater risk of bone fractures. While physical activity and intake of both calcium and vitamin D benefit BMD in older children, there is limited research on the determinants of good bone health in early childhood. The aim of this cross-sectional study was to investigate the impact of diet, physical activity, and body composition on BMD at five years of age. Dietary intakes and physical activity levels were measured through questionnaires. Whole body BMD was measured by dual-energy X-ray absorptiometry in 102 children. Child weight, height, circumferences, skinfolds and serum 25-hydroxyvitamin D (25OHD) concentrations were assessed. There was no association between BMD and dietary calcium, dietary vitamin D, 25OHD, physical activity, or sedentary behaviour. Several measures of body composition were significantly positively associated with BMD; however, neither fat mass nor lean body mass was associated with BMD.

*Conclusion*: Although we found no association between self-reported dietary and lifestyle factors and bone health in early years, increased body size was linked with higher BMD. These findings are important as identifying modifiable factors that can improve bone health at a young age is of utmost importance.

**Table:** 

**What is Known:** • *Bone health is extremely important in early childhood, as children with low bone mineral density (BMD) are at greater risk of bone fractures*. • *Physical activity has been found to be beneficial for bone health in adolescents, and body composition has also been associated with BMD in teenage years*. • *Limited research on the determinants of good bone health in early childhood*.	
**What is New:** • *No association between self-reported lifestyle and dietary factors with bone health in early childhood*. • *Increased body size was associated with higher BMD at five years of age*.

**What is Known:**

• *Bone health is extremely important in early childhood, as children with low bone mineral density (BMD) are at greater risk of bone fractures*.

• *Physical activity has been found to be beneficial for bone health in adolescents, and body composition has also been associated with BMD in teenage years*.

• *Limited research on the determinants of good bone health in early childhood*.

**What is New:**

• *No association between self-reported lifestyle and dietary factors with bone health in early childhood*.

• *Increased body size was associated with higher BMD at five years of age*.

## Introduction

During normal childhood and adolescence, the skeleton undergoes tremendous change. Unlike the adult skeleton where bone remodelling is the dominant activity, the younger skeleton is both growing and remodelling. It is estimated that more than half of peak bone mass is acquired during the teenage years [1]. Thus, factors that influence bone health during early life are extremely important. Dual-energy X-ray absorptiometry (DXA) is the best measure of bone health by estimating areal bone mineral density (BMD) and areal bone mineral content (BMC) [28]. Children with low BMD are at higher risk of fractures throughout childhood [7]. Individuals with low BMD in childhood who have concomitant multiple bone fractures are also at a higher risk of being diagnosed with osteoporosis in later life [21]. In order to delay the onset of osteoporosis, it is vital to ensure that BMD is at an optimum level throughout childhood and adolescence.

Heredity is the principal determinant of BMD [10, 22, 36], but identifying modifiable factors has the potential for benefit. The relationship between bone health and diet is well established, particularly focusing on calcium and vitamin D [23, 30, 41–43]. Since effective sunlight exposure in Ireland is curtailed to 7 months yearly, oral intake of vitamin D from all sources (natural foodstuffs, fortified foods and supplements) is essential to maintain serum 25-hydroxyvitamin D (25OHD) levels year round [6]. Calcium intake in Ireland tends to be at the high end of the international spectrum in adults in view of the ready availability of dairy products but intake can vary widely [3]. Physical activity has a positive association with BMD in children and adolescents [5, 16, 44]. An intervention study demonstrated that a school-based jumping programme significantly increased BMD in prepubertal boys and girls [31, 32].

The association between body composition and bone health is conflicting. In adults, BMD had a stronger relationship with lean body mass than with fat mass [19, 39]. It has been suggested that adipose tissue is not beneficial to bone structure in healthy mature adolescents and young adults [18, 34]. However, in prepubertal children, it is proposed that adipose tissue may stimulate bone growth [8], and it has been reported that fat mass was positively associated with bone mass in children [24, 38].

The aim of this research project is to provide insight into modifiable factors that may be related to bone health in preschool-aged Irish children. Based on secondary analysis of data from the ROLO Kids Study, we aimed to investigate the associations between diet, physical activity and body composition with child bone mineral density in 5-year-old children.

## Methods

### Study design and population

The ROLO Study was a randomised controlled trial of a low glycaemic index diet during pregnancy, with the aim of reducing foetal macrosomia. In the study, 800 mothers were recruited that had previously given birth to a macrosomic child (birth weight > 4 kg) and had no adverse health conditions. Mothers were randomised into an intervention or control group, where the intervention group received low glycaemic index dietary advice to follow during pregnancy, and the control arm had routine antenatal care. Findings from the ROLO Study and follow-up studies have previously been published [14, 29, 40], but in brief, there was no significant difference in birth weight between the control and intervention groups. Women in the intervention group did, however, reduce their gestational weight gain and improve their glycaemic control. The mothers and infants from this study were then followed up as part of the longitudinal ROLO Kids Study. Mothers were contacted and invited to take part in this analysis when their child turned 5 years of age. Written maternal consent was obtained at the follow-up visit and this study received ethical approval by the Ethics Committee in Our Lady’s Children Hospital, Dublin. Participants were eligible for this analysis if they had taken part in the ROLO Kids Study and had a DXA scan as part of the follow-up. A total of 102 mother and child dyads were included in this secondary analysis.

### BMD and body composition measurements

BMD of the mother and child was measured by DXA using the Lunar iDXA^TM^ scanner (GE Healthcare, Madison WI) with enCORE^TM^ v.14.1 software. Measurements obtained by DXA included BMD (g/cm^2^), fat mass (kg) and lean mass (kg). Fat mass as a percentage of whole-body tissue was derived. In order to minimise the effect of operator variability factor, one or other of two qualified observers analysed all scans at 5 years follow-up. In terms of clinically relevant findings, based on a sample size calculation, to detect 1 standard deviation (SD) difference in BMD in our cohort, 17 participants are required per group (e.g. 17 males, 17 females) for 80% power at 0.05 level of significance.

### Dietary measurements

The children’s mothers completed a food frequency questionnaire (FFQ) on behalf of their child. This measured their child’s habitual dietary intakes over the previous year. The FFQ was divided into different categories—breads/pastas, cakes/confectionary, dairy, sugar sweetened beverages, fruit/vegetables, spreads and meat/fish—and asked mothers to report how frequently their child consumed each food product in the last year. The FFQ was based on the Growing up in Ireland 5-Year Primary Caregiver Main Questionnaire [12] and was a validated method of assessing dietary intakes in children. Using McCance and Widdowson’s *The Composition of Foods Seventh Summary Edition* [35], habitual dietary intakes of calcium and vitamin D were calculated in milligram per day (mg/day) and microgram per day (μg/day), respectively.

### Physical activity and sedentary behaviour

Mothers also completed the Children’s Leisure and Activity Study Survey (CLASS) questionnaire on behalf of their child. The CLASS questionnaire is a baseline proxy report of typical week physical activity and sedentary behaviour questionnaire. This is a validated questionnaire estimating habitual physical activity and sedentary activity levels in children [17]. The CLASS questionnaire also estimated the amount of screen time the child had each week, which was used as a proxy for sedentary behaviour.

### Anthropometric measurements

The height and weight of the child was measured using a SECA stadiometer (to the nearest 0.1 cm) and a SECA scales (to the nearest 0.1 kg), respectively, and body mass index (BMI) was calculated as kilogram per square metre (kg/m^2^), along with standardised scores and centiles, which was scored using the definitions set by the Centre for Disease Control and Prevention [4]. These measurements and calculations were all carried out by the trained research team. Circumferences of the head, neck, mid-upper arm (MUA), chest, abdomen, hip and thigh were measured using a SECA ergonomic circumference measuring tape (to the nearest 0.1 cm). Skinfold measurements of the biceps, triceps, subscapular and thigh were collected (to the nearest 0.2 mm) using a Holtain skinfold caliper, and subscapular to triceps ratio, sum of skinfolds and subscapular and triceps skinfold thicknesses as markers of adiposity were calculated.

### 25OHD measurement

Blood samples (*n* = 32) were collected at the follow-up visit by a trained phlebotomist. 25OHD concentrations were quantified using the Elecsys Vitamin D Total (Roche Diagnostics GmbH, Mannheim, Germany) automated competitive binding protein assay.

### Statistical analysis

The distribution and normality of each variable was determined by visual assessment of histograms. Mean and SD were reported for normally distributed data with median and interquartile range (IQR) being reported for non-normal data. To measure unadjusted correlations between each variable and the child’s BMD, Pearson’s correlation was used for normally distributed data and Spearman’s correlation for non-normal data to explore the relationship. A significance level of *p* < 0.05 was used to set a cut-off for further analysis with each variable. Multiple regression analysis was then used to identify the extent of the relationship between child BMD and each variable with a significant correlation, while controlling the following confounders: child sex, maternal BMD, maternal education level (as a marker of socioeconomic status) and whether the child was breastfed or not. Each variable was run individually with its own model, with 12 models being created in total. The population was dichotomized based on child’s adherence to recommended nutrient intakes (> 800 mg/day calcium, > 5 μg/day vitamin D, optimal serum 25OHD status (> 50 nmol/L), physical activity (> 420 min/week), screen time (< 1 h/day) and BMI normal weight status (< 85th percentile). Differences in BMD between these groups were assessed using independent sample *t* tests for normally distributed data and Mann-Whitney*U* tests for non-normal data. All analysis was carried out using the Statistical Package for the Social Sciences (SPSS) version 24 (SPSS Inc., Chicago, USA).

## Results

### Descriptive statistics

The mean age of participants in this cohort was 5.09 years (51% female) and they were all Caucasian, with one being of Asian descent, with highly educated mothers, over 60% having completed third-level education (Table 1). There was no significant difference in any potential confounding factors between the sexes; however, there was a significant difference in BMD with males having a significantly higher BMD than females (*p* < 0.01).

**Table 1:** Descriptive statistics of the participants in the ROLO Kids Study

	Total			Male			Female			*p*
	*n*			*n*			*n*			
Age at follow-up (mean, SD, years)	102	5.09	0.13	47	5.07	0.13	55	5.1	0.13	0.167
Ethnicity (Caucasian (%))	102	101 (99)		47	46 (97.9)		55	55 (100)		0.277
RCT group (intervention, *n* (%))	102	49 (48)		47	23 (48.9)		55	26 (47.3)		0.867
Maternal education (achieved 3rd level (%))	94	60 (63.8)		44	29 (65.9)		50	31 (62)		0.694
Breastfed status (yes (%))	99	70 (70.7)		47	32 (68.1)		52	38 (73.1)		0.586
BMD (mean, SD, g/cm^2^)	102	0.676	0.05	47	0.689	0.04	55	0.665	0.05	0.006*

*SD*, standard deviation; *RCT*, randomised control trial; *BMD*, bone mineral density

*Correlation is significant at 0.05 level (2-tailed)

Comparing between males and females, there was no difference in calcium or vitamin D intake, 25OHD concentration, physical/sedentary activity or body mass index (BMI)(Table 2). However, there were differences in body composition; male children had significantly higher head, neck and chest circumferences than females, while females had higher thigh circumferences and central adiposity measurements (*p* < 0.01).

**Table 2:** Descriptive statistics for dietary intakes, physical activity and body composition

	Total			Male			Female			*p*
	*n*			*n*			*n*			
Diet and 25OHD status										
Energy (mean, SD, kcal)	97	1,248.77	445.20	46	1,338.13	30.98	51	1168.18	418.98	0.276
Protein (median, IQR, g)	97	51.87	28.30	46	55.36	30.98	51	50.85	29.98	0.094
Fat (mean, SD, g)	97	41.76	15.88	46	44.01	14.61	51	39.73	16.84	0.186
Carbohydrate (median, IQR, g)	97	158.14	62.09	46	166.68	47.11	51	140.6	82.57	0.048*
Calcium (mean, SD, mg)	97	824.67	356.38	46	866.33	346.32	51	787.07	364.52	0.276
Vitamin D (median, IQR, μg)	97	1.83	1.64	46	1.8	1.93	51	1.9	1.34	0.931
Fibre (mean, SD, g)	97	11.97	5.68	46	12.57	6.2	51	11.43	5.18	0.323
Serum 25OHD (median, IQR, nmol/L)	32	52.25	24.73	20	52.9	34.95	12	52.25	18.43	0.863
Physical activity										
Moderate PA (median, IQR, min/week)	73	300	250	36	300	257.5	37	300	242	0.873
Moderate PA (median, IQR, METs/week)	73	1380	1136	36	1379	1228.25	37	1380	1121	0.762
Vigorous PA (median, IQR, min/week)	73	175	195	36	210	178.75	37	150	212.5	0.216
Vigorous PA (median, IQR, METs^a^/week)	73	2343	8778	36	3372.5	7611.5	37	1604	11,593	0.284
Total PA (median, IQR, min/week)	73	500	380	36	523.5	371.25	37	495	328	0.456
Screen time (median, IQR, min/week)	73	600	540	35	720	600	37	600	535	0.054
Body composition										
Weight (mean, SD, kg)	102	19.99	2.41	47	20.2	2.05	55	19.82	2.7	0.436
Length (mean, SD, cm)	102	111.12	4.26	47	111.51	3.89	55	110.79	4.56	0.395
BMI (mean, SD, kg/m^2^)	102	16.15	1.25	47	16.22	1.06	55	16.09	1.4	0.621
Weight centile (median, IQR)	102	61.5	45	47	66	40	55	56	46	0.314
Length centile (median, IQR)	102	60	53	47	60	50	55	60	57	0.689
BMI centile (median, IQR)	102	66.5	42	47	72	44	55	61	41	0.290
Head circ. (mean, SD, cm)	102	51.64	1.44	47	51.98	1.44	55	51.35	1.39	0.026*
Neck circ. (mean, SD, cm)	102	25.29	1.36	47	25.81	1.29	55	24.85	1.26	< 0.010*
MUA circ. (mean, SD, cm)	102	17.52	1.27	47	17.45	1.11	55	17.59	1.4	0.591
Chest circ. (mean, SD, cm)	101	56.22	2.63	47	56.92	2.04	54	55.61	2.93	0.012*
Abdomen circ. (mean, SD, cm)	102	54.53	4.97	47	54.42	5.64	55	54.62	4.37	0.844
Hip circ. (mean, SD, cm)	102	59.6	4.08	47	59.72	3.59	55	59.49	4.48	0.772
Thigh circ. (mean, SD, cm)	102	32.54	3.05	47	31.85	2.87	55	33.13	3.1	0.034*
Triceps skinfold (mean, SD, cm)	100	9.84	2.51	47	9.36	2.56	53	10.27	2.4	0.071
Biceps skinfold (mean, SD, cm)	101	5.36	1.56	47	5.21	1.54	54	5.50	1.57	0.344
Subscapular skinfold (mean, SD, cm)	99	5.94	1.93	47	5.6	1.22	52	6.26	2.36	0.089
Thigh skinfold (mean, SD, cm)	100	15.02	4.99	47	14.23	5.18	53	15.72	4.77	0.139
General adiposity (mean, SD, cm)	99	36.17	9.35	47	34.4	9.18	52	37.77	9.30	0.073
Central adiposity (mean, SD, cm)	99	15.79	3.96	47	14.96	3.56	52	16.54	4.18	0.047*
Subscap to triceps ratio (median, IQR, )	99	0.6	0.17	47	0.61	0.13	52	0.59	0.20	0.473
Lean body mass (mean, SD, kg)	102	14.45	1.55	47	14.48	1.36	55	14.42	1.71	0.825
Fat mass (mean, SD, kg)	102	4.81	1.28	47	4.54	1.31	55	5.03	1.21	0.019*
Lean body mass percentage (mean, SD)	102	72.5	4.6	47	73.82	5.1	55	71.37	3.76	< 0.010*
Fat mass percentage (mean, SD)	102	23.81	4.35	47	22.83	4.69	55	24.65	3.88	0.022*

*SD*, standard deviation; *IQR*, interquartile range; *PA*, physical activity; *METs*, metabolic equivalents; *BMI*, body mass index; *MUA*, mid-upper arm

IQR reported as a single value (calculated as the 75th percentile–25th percentile)

*p is significant at *p* < 0.05.

The mean calcium intake across the population was 824 mg with 866 mg and 787 mg being the mean intake for male and female participants, respectively. The median vitamin D intake was 1.83 μg with similar intakes found between males and females (*p* = 0.931) Reported physical activity and screen time levels were 500 min/week and 600 min/week, respectively.

### Achieving recommended intakes

Almost half of the participants met the Irish recommended daily allowance for calcium intake for children of 800 mg/day [3], whereas only 6% had an adequate vitamin D intake of 5 ug/day(Table 3) [13]. Over 68% of the children were reported to exercise for more than 420 min/week [11] but just over a quarter had the recommended less than 1 hour of screen time/day [9]. Over 80% of the study population were within the ranges for a healthy BMI centile (< 85^th^ centile) with almost 20% being in the overweight (85th–95th) or obese category (> 95th BMI centile).

**Table 3:** Proportion of population meeting recommendations for diet, physical and sedentary behaviour and body composition

	Total		Male		Female	
	*n*		*n*		*n*	
Diet and 25OHD status						
> 800 mg calcium (*n* (%))^1^	97	46 (47.4)	46	23 (50)	51	23 (45.1)
> 5 μg vitamin D (*n* (%))^1^	97	6 (6.2)	46	3 (6.5)	51	3 (5.9)
> 50 nmol/L 25OHD concentration (*n* (%))^2^	32	20 (62.5)	20	11 (55)	12	9 (75)
Physical and sedentary behaviour						
> 420 min total PA/week (*n* (%))^3^	73	50 (68.5)	36	26 (72.2)	37	24 (64.9)
< 1 h screen time/day (*n* (%))^4^	72	19 (26.4)	35	7 (20)	37	12 (32.4)
Body composition						
Normal (BMI < 85th centile) (*n* (%)) ^5^	102	82 (80.4)	47	37 (78.7)	52	45 (81.8)
Overweight (85th < BMI < 95th centile) (*n* (%)) ^5^	102	12 (11.8)	47	7 (14.9)	52	5 (9.1)
Obese (BMI > 95th centile) (*n* (%)) ^5^	102	8 (7.8)	47	3 (6.4)	52	5 (9.1)

*25OHD*, serum vitamin D; *PA*, physical activity; *BMI*, body mass index

^1^Scientific guidelines for healthy eating in Ireland (2011)

^2^IOM (2011)

^3^The national guidelines on physical activity for Ireland (2009)

^4^Media use in school-aged children and adolescents: AAP (2016)

^5^Centres for Disease Control and Protection, Defining childhood obesity (2016)

### Associations with BMD

Neither meeting calcium recommendations having adequate 25OHD concentrations, nor meeting physical activity recommendations was significantly associated with BMD at 5 years of age (*p* > 0.05, Table 4). BMD was significantly lower in children who were in the normal weight category (< 85th BMI centile) than those who were in the overweight category (> 85th BMI centile) (*p* < 0.01). When the participants were split into male and female categories, this relationship with BMD and BMI centile was only seen in female participants (*p* < 0.01).

**Table 4:** Meeting current recommendations and differences in bone mineral density in the study population

	Total			Male			Female		
	*n*	BMD (g/cm^2^)	*p*	*n*	BMD (g/cm^2^)	*p*	*n*	BMD (g/cm^2^)	*p*
Diet									
> 800 mg calcium	46	0.674	0.942	23	0.683	0.402	23	0.664	0.737
< 800 mg calcium	51	0.674		23	0.693		28	0.659	
> 5 μg vitamin D	6	0.631	0.014*	3	0.672	0.443	3	0.589	< 0.010*
< 5 μg vitamin D	91	0.677		43	0.689		3	0.666	
> 50 nmol/L 25OHD	20	0.675	0.400	11	0.689	0.297	9	0.659	0.600
< 50 nmol/L 25OHD	12	0.690		9	0.706		3	0.641	
Physical activity									
> 420 min PA/week	50	0.678	0.795	26	0.693	0.557	24	0.661	0.746
< 420 min PA/week	23	0.675		10	0.686		13	0.667	
< 1 h screen time	19	0.663	0.173	7	0.694	0.842	12	0.645	0.186
> 1 h screen time	53	0.679		28	0.691		25	0.666	
Body composition									
BMI < 85th centile	81	0.668	< 0.010*	36	0.687	0.381	45	0.654	< 0.010*
BMI > 85th centile	21	0.705		11	0.698		10	0.712	

*25OHD*, serum vitamin D concentration; *PA*, physical activity; *BMI*, body mass index

*p is significant at *p* < 0.05, performed using Mann-Whitney *U* tests

### Unadjusted correlations

All dietary factors, physical and sedentary activity and anthropometric measurements were then correlated with BMD, for the total group and according to each sex (Table 5). In the total group, no dietary factors were significantly correlated with BMD. Physical activity was not significantly correlated with BMD; however, body composition was significantly correlated with BMD at five years of age. Weight (*p* < 0.01), length (*p* < 0.05), BMI, weight centile and BMI centile (all *p* < 0.01), and all circumferences were positively correlated with BMD. Although triceps skinfold (*p* < 0.01 = 0.000), subscapular skinfold (*p* < 0.01), sum of skinfolds (*p* < 0.01) and triceps and subscapular skinfold (*p* < 0.01) were positively correlated with BMD in females, no skinfold or adiposity measurement was significantly associated with BMD in the total population.

**Table 5:** Unadjusted correlations of factors with bone mineral density in 5 years old children

	Total		Male		Female	
	*r*	*p*	*r*	*p*	*r*	*p*
Diet						
Calcium (mg)	0.055	0.595	− 0.163	0.281	0.144	0.314
Vitamin D (μg)	− 0.173	0.090	− 0.330	0.025*	− 0.099	0.486
Energy (kcal)	− 0.084	0.413	− 0.357	0.015*	0.003	0.985
Protein (g)	0.004	0.966	− 0.15	0.319	0.055	0.701
Carbohydrate (g)	− 0.014	0.170	− 0.424	0.003**	− 0.067	0.638
Fat (g)	0.042	0.686	− 0.164	0.275	0.101	0.482
Fibre (g)	− 0.114	0.263	− 0.345	0.019*	< 0.001	0.997
Serum 25OHD (nmol/L)	0.161	0.396	− 0.286	0.222	< 0.001	1.000
Physical activity						
Moderate PA (min/week)	0.017	0.888	0.201	2.240	− 0.156	.358
Moderate PA (METs/week)	0.001	0.991	0.210	0.219	− 0.186	0.269
Vigorous PA (min/week)	− 0.017	0.889	− 0.154	0.369	− 0.037	− 0.084
Vigorous PA (METs/week)	0.005	0.970	0.081	0.640	− 0.084	0.622
Screen time (min/week)	0.111	0.354	0.016	0.926	0.057	0.736
Body composition						
Weight (kg)	0.409	0.000**	0.03	0.842	0.591	0.000**
Length (cm)	0.248	0.012*	− 0.11	0.460	0.429	0.000**
BMI (kg/m^2^)	0.385	0.000**	0.151	0.310	0.505	0.000**
Weight centile	0.365	0.000**	− 0.035	0.816	0.579	0.000**
Length centile	0.190	0.056	− 0.157	0.293	0.390	0.003**
BMI centile	0.384	0.000**	0.128	0.391	0.499	0.000**
Head circ. (cm)	0.392	0.000**	0.306	0.037*	0.393	0.003**
Neck circ. (cm)	0.437	0.000**	0.215	0.146	0.495	0.000**
MUA circ. (cm)	0.347	0.000**	0.039	0.792	0.552	0.000**
Chest circ. (cm)	0.294	0.003**	− 0.040	0.791	0.373	0.005**
Abdomen circ. (cm)	0.199	0.044*	0.055	0.711	0.360	0.007**
Hip circ. (cm)	0.248	0.012*	− 0.077	0.607	0.423	0.001**
Thigh circ. (cm)	0.195	0.050*	− 0.047	0.753	0.462	0.000**
Triceps skinfold (cm)	0.174	0.083	− 0.084	0.575	0.477	0.000**
Biceps skinfold (cm)	0.058	0.562	− 0.142	0.341	0.238	0.083
Subscapular skinfold (cm)	0.148	0.145	0.019	0.897	0.28	0.044*
Thigh skinfold (cm)	0.082	0.419	0.079	0.597	0.163	0.245
Sum of skinfolds (cm)	0.126	0.213	0.000	1.000	.0320	0.021*
Subscapular and triceps skinfold (cm)	0.184	0.069	− 0.054	0.720	0.443	0.001**
Subscap to triceps ratio	0.004	0.965	0.088	0.555	− 0.088	0.534
Lean body mass (kg)	− 0.075	0.453	0.035	0.818	− 0.145	0.290
Fat mass (kg)	− 0.16	0.108	0.095	0.524	− 0.266	0.050*
Lean body mass percentage	0.156	0.117	− 0.098	0.511	− 0.266	0.050*
Fat mass percentage	− 0.113	0.260	0.116	0.439	− 0.2	0.143

*PA*, physical activity; *METs*, metabolic equivalents; *BMI*; body mass index; *circ*.: circumference; *MUA*, mid-upper arm

*Correlation is significant at the 0.05 level (2-tailed)

**Correlation is significant at the 0.01 level (2-tailed)

### Multiple regression analysis

In total, 12 separate multiple regression models were created to estimate BMD using measures of body composition, all of which are summarised in Table 6. Apart from abdominal circumference, lean body mass and fat mass, all body composition measurements were significant at *p* < 0.05 level, when all confounding factors were controlled.

**Table 6:** Multiple regression analysis models for bone mineral density in 5 years old children

		*b*	*p*	95% CI Lower	95% CI Upper	Adjusted *r*^2^	*F*	*p*
Model 1								
	BMI (kg/m^2^)^a^	0.013	< 0.001*	0.007	0.019	0.292	7.183	< 0.001
Model 2								
	Weight (kg)^a^	0.007	< 0.001*	0.004	0.01	0.294	7.24	< 0.001
Model 3								
	Length (cm)^a^	0.002	0.042*	0.000	0.004	0.18	4.292	0.001
Model 4								
	Head circ. (cm)^a^	0.01	0.004*	0.004	0.015	0.247	5.928	< 0.001
Model 5								
	Neck circ. (cm)^a^	0.012	< 0.001*	0.005	0.018	0.259	6.25	< 0.001
Model 6								
	MUA circ. (cm)^a^	0.012	< 0.001*	0.006	0.019	0.267	6.475	< 0.001
Model 7								
	Chest circ. (cm)^a^	0.004	0.023*	0.001	0.007	0.19	4.489	0.001
Model 8								
	Thigh circ. (cm)^a^	0.005	0.002*	0.002	0.007	0.231	5.499	< 0.001
Model 9								
	Hip circ. (cm) ^a^	0.003	0.011*	0.001	0.005	0.202	4.808	< 0.001
Model 10								
	Abdominal circ. (cm)^a^	0.001	0.144	0.000	0.003	0.16	3.862	0.002
Model 11								
	Lean body mass (kg)^a^	− 0.002	0.391	− 0.008	0.003	0.146	3.566	0.003
Model 12								
	Fat mass (kg)^a^	− 0.003	0.332	− 0.01	0.004	0.148	3.61	0.003

*BMI*; body mass index; *circ*: circumference; *MUA*; mid-upper arm

^a^Model adjusted for child sex, maternal BMD, maternal education level, membership of intervention/control group, breastfeeding status

*Regression is significant at *p* < 0.05

## Discussion

In this study, we aimed to investigate the associations of diet, physical activity and body composition with child BMD at 5 years of age. For dietary intakes, there was no evidence for an association with BMD at 5 years of age. Similarly, no evidence was found for an association between parental-reported physical activity levels or sedentary behaviour on child BMD. However, we found strong evidence for an association between body composition at 5 years of age and child BMD, particularly with weight, child BMI and body circumferences.

According to a recent American study, the proportion of 4–8 years old children meeting the 800 mg/day recommendation of calcium is 80% in boys and 67% in girls [2]. Our findings suggest that Irish children are behind their American counterparts for calcium intake. However, after splitting the population into those who met and did not meet the recommended 800 mg (47% vs 53%), we found no significant difference in BMD between these two groups. Other studies have, however, reported an association between calcium supplementation and BMD [23, 25]. Similar results were seen in relation to vitamin D intake. The frequency of adequate dietary intake of vitamin D was much lower than calcium intake, with only 6% of the study cohort meeting recommendations of a daily intake of 5 μg. This is not uncommon in young children; European reports estimate that up to 40% of children have inadequate D status [6]. Although vitamin D supplementation has shown no effect on BMD in children with adequate vitamin D intakes [41, 42], an on-going study in vitamin D-deficient children should provide an interesting outlook on vitamin D’s effect on child bone health [43]. Taking this into account, further research into habitual dietary calcium and vitamin D intakes and bone health in young children is required.

While we found no association between physical activity levels and child BMD, intervention studies which implemented an exercise activity or studies which compared children who engage in a competitive sport with untrained controls showed significant associations between physical activity and higher BMD [37, 44]. As our population had a high level of physical activity, this may make it difficult to identify associations. Other research indicates how important physical activity can be when looking to optimise bone accrual and the importance of regular exercise during childhood cannot be dismissed. We also found that parental-reported sedentary behaviour had no association with BMD, which is comparable with current research. Recent studies suggested that only objectively measured sedentary time, using methods like accelerometers, was negatively associated with lower extremity bone outcomes in schoolchildren and that subjectively measured sedentary time (use of questionnaires) had no association with bone outcomes [20]. Although our results are in line with current research, it’s important to note that this relates to screen time as a measure of sedentary behaviour. Other characteristics of sedentary behaviour such as being in a sitting, reclining or lying posture may also have a significant effect on BMD. Even though sedentary behaviour was not strongly associated with a decrease in bone health, and our findings suggest no association between physical activity and BMD, interventions in other studies have shown how influential physical activity can be to bone health [26, 27]. Therefore, replacing sedentary activities with increased physical activity during childhood and adolescence could prove to have a prolific effect on bone health and aid the optimisation of bone accrual amidst this crucial growth phase.

We found that child body composition at 5 years of age was significantly associated with BMD. Eighty percent of our cohort was classified as having a healthy BMI. These findings are parallel with recent Irish figures, which estimate that 1 in 4 children is overweight or has obesity in Ireland [33], and US figures, stating that 16% of 2–5-year-old children are overweight or have obesity [15]. Results from our study support the theory that increased body mass leads to increased bone strength, since weight, BMI, weight centile and BMI centile were all significantly positively correlated with BMD (all *p* < 0.001) and remained statistically significant when all confounders were controlled. Although overall weight and circumferences had a significant association with child BMD, neither lean body mass nor fat mass was associated. Current research investigating the impact of fat mass on BMD is conflicting, while lean body mass has been shown to have a positive relationship with bone health [19, 39]. Previous studies have concluded that adipose tissue was not beneficial to bone structure [18, 34], while another study suggested that fat mass is on the causal pathway, having a positive relationship with bone mass in children [38].

This study had many strengths in that we used DXA to measure the children’s bone density as this is the gold standard of measuring bone density. We also used validated questionnaires to measure physical activity and screen time levels [17]. Another major strength of this study was the intentional recruitment of children within a very narrow age range (4.83–5.5 years old) which allowed us to investigate bone health at this time point without too much variation. One potential limitation is the sample size of 102 participants in this study. While it would have been interesting to investigate the impact of ethnicity on bone health, 99% of participants in this analysis were Caucasian, and further research is required in other ethnic groups. Another limitation is the use of a food frequency questionnaire to estimate micronutrient status, because a more detailed measure, such as a food diary, would be more appropriate and this should be considered for future research investigating associations with vitamin D and calcium. A final potential limitation to this study is that the information on the season that the blood samples were drawn was not noted and therefore any association made between 25OHD levels and BMD may be compromised.

Future research should be carried out in children of similar age, as the current volume of research on modifiable factors impacting bone health at this age is quite limited. Although there are a multitude of such studies conducted in preadolescents and older children, there is a dearth in studies in children in early childhood which is a vital time of growth and development.

We found that increased body size was associated with higher BMD. As this relationship was identified predominantly in the female cohort and was weaker when analysing males only, further research is warranted in this area in order to identify the potential differential effect of sex on BMD during childhood. We identified no association between self-reported lifestyle and dietary factors and bone health in early childhood as measured by BMD analysis using DXA. This suggests that in early childhood body weight may be an important indicator of child bone health. Thus, child body size could potentially be identified as a modifiable factor in interventions aiming to improve bone health, and monitoring body size throughout childhood may also be beneficial in terms of bone health. This is important for bone health in later life as optimising BMD during childhood can reduce the risk of fractures in adolescence and adult life. Identifying modifiable factors that can improve bone health at a young age is of utmost importance. Further research is required in this area to elucidate the exact mechanisms whereby body weight can influence bone health and to identify methods of ensuring children reach peak bone mass for optimal bone health later in life.

## Abbreviations

25OHD: 25-hydroxyvitamin D
BMC: Bone mineral content
BMD: Bone mineral density
BMI: Body mass index
CLASS: Children’s Leisure and Activities Study Survey
DXA: Dual-energy X-ray absorptiometry
FFQ: Food frequency questionnaire
IQR: Interquartile range
MUA: Mid-upper arm
RDA: Recommended daily allowance
SD: Standard deviation
